# Navigating the Mpox Outbreak: Insights on Vaccination Decisions and Psychosocial Impacts Among Gay, Bisexual, and Other Men Who Have Sex with Men in Los Angeles

**DOI:** 10.1007/s10461-025-04916-3

**Published:** 2025-11-09

**Authors:** Elizabeth A. Yonko, Connor G. Wright, Lauren Rabbottini, Kiana Aminzadeh, Kenneth H. Mayer, Katie B. Biello, Matthew J. Mimiaga

**Affiliations:** 1 Department of Epidemiology, UCLA Fielding School of Public Health, Los Angeles, CA USA; 2 UCLA Center for LGBTQ+ Advocacy, Research and Health, Los Angeles, CA USA; 3 School of Social Work, Boston University, Boston, MA USA; 4 The Fenway Institute, Fenway Health, Boston, MA USA; 5 Department of Medicine, Harvard Medical School/Beth Israel Deaconess Medical Center, Boston, MA USA; 6 Department of Epidemiology, Brown University School of Public Health, Providence, RI USA; 7 Department of Psychiatry and Biobehavioral Sciences, UCLA Geffen School of Medicine, Los Angeles, CA USA

**Keywords:** Mpox, Monkeypox, Mpox vaccination, HIV, COVID-19, Gay and bisexual men, Men who have sex with men, MSM, Mixed methods

## Abstract

The 2022 mpox outbreak significantly impacted gay, bisexual, and other men who have sex with men (MSM) in the U.S., with Los Angeles representing nearly 40% of California cases. Limited data exist on how MSM living with and without HIV navigated the outbreak and their decision-making regarding vaccination. Between November 2023 and March 2024, we conducted a mixed-methods study with 21 cisgender MSM in Los Angeles who completed a semi-structured interview and quantitative survey. Recruitment prioritized diversity in mpox vaccination history, HIV status, race, and ethnicity. Interviews were recorded, transcribed, and analyzed using thematic content analysis; surveys were analyzed using descriptive statistics. Participants had a mean age of 40 years; 33% were living with HIV; 48% identified as Black/African American or mixed-race, 38% White; nearly half identified as Hispanic/Latinx (48%); 57% reported receiving at least one mpox vaccine dose, and 38% reported two doses for maximum protection. Knowledge about mpox transmission, prevention, treatment, and outcomes varied. Participants self-reported hearing that mpox could be transmitted by skin-skin contact (76%), kissing (67%), engaging in oral sex (67%), and contact with semen (33%); 62% had heard there was a treatment for mpox, 14% thought that mpox was not curable, and 48% believed that mpox was likely to cause death. Psychosocial impacts were prominent at both individual and community levels, including fear and distress. Mpox vaccination was motivated by fear and a desire for protection, facilitated by accessible venues and peer influence. Barriers included initially poor availability of vaccine and the specific eligibility criteria requirements for vaccination early on in the epidemic. Logistical challenges, such as long wait times (> 2 h), work hours, lack of transportation, mistrust in research, and confusing initial rollout also presented unique barriers. LGBTQ+ community-based organizations and peer networks were the primary trusted source of mpox-related information. Most initially adopted risk reduction behaviors but generally viewed the mpox response more favorably than COVID-19 due to time differences in vaccine availability. Post-vaccination, many resumed pre-outbreak activities, feeling more prepared for future outbreaks despite some lingering concerns. Findings underscore varied mpox knowledge and significant psychosocial impacts, reminiscent of the early HIV epidemic. Key facilitators and barriers to vaccination highlight the critical reliance on LGBTQ+ community-based organizations and peer networks for sources of trustworthy information. Providing referrals to mental health counseling and other forms of support during vaccination is recommended.

## Introduction

Mpox, previously known as monkeypox, is an *Orthopoxvirus* that was declared a public health emergency by the World Health Organization (WHO) on July 23, 2022, following significant transmission of the Clade IIb lineage in historically non-endemic regions [1]. This outbreak primarily affected gay, bisexual, and other men who have sex with men (MSM), with transmission largely occurring through close skin-to-skin contact in sexual settings [2]. As of January 10th, 2024, the United States (U.S.) has reported more than 32,000 confirmed cases, accounting for more than a third of the global total [3, 4]. California reported the highest number of cases in the U.S., with 6,476 laboratory-confirmed infections, and Los Angeles County alone representing nearly 40% of cases statewide [5]. Los Angeles is home to one of the largest and most vibrant LGBTQ+ communities in the U.S., with an estimated 9% of adults identifying as LGBTQ+ in Los Angeles County [6]. Los Angeles also has one of the largest number of queer venues (i.e., bars and dance clubs, sex clubs and bathhouses), and these spaces played a notable role in both mpox transmission and community-driven public health responses [7].

The domestic public health response to mpox has been spearheaded by the U.S. Centers for Disease Control and Prevention (CDC), which implemented adapted surveillance systems, diagnostic tests, vaccines, therapeutics, grants, and communication strategies [2]. Community activism and engagement have been crucial to the success of the mpox response. Early warnings about testing capacity, vaccine availability, and treatment access prompting the government to prioritize its domestic response [8]. Within three months of the first reported case, messaging campaigns were launched, and more than 1 million vaccine doses were distributed, contributing to a decline in caseloads [4].

Mpox significantly impacts the physical well-being of those affected. The most common symptom is the appearance of pox-like lesions, which can lead to complications such as proctitis, tonsillitis, and skin and soft tissue infections [9]. These symptoms are often reported as extremely painful and may necessitate hospitalization for severe rectal pain and penile swelling [10]. Mpox lesions typically take 2 to 4 weeks to heal and can result in considerable scarring in the affected areas [11, 12]. While few cases have been fatal, individuals with advanced HIV (i.e., immunocompromised) face an increased risk of severe illness and death if they contract mpox [3, 13].

The clinical impact of mpox extends beyond its immediate health effects. Research from endemic regions highlights not only the short-term consequences of the virus on patients but also concerns regarding long-term stigmatization and social isolation [14]. A systematic review of studies conducted during the outbreak found that 25 to 50% of mpox patients experience anxiety and depression, influenced by factors such as shame over physical symptoms (e.g., scarring), fear of stigmatization, and concerns about transmitting the infection [15]. While treatments for mpox are available, there is limited understanding of the mental health impact beyond the recovery phase [16]. This gap leaves us with an incomplete picture of the lasting psychosocial effects of mpox, particularly among communities most affected, including MSM and people living with HIV (PLWH).

The U.S. response to the mpox outbreak included both formalized public health messaging and more informal community mobilization. However, there is a lack of peer-reviewed evaluations regarding the acceptability of these approaches. In the United Kingdom, findings indicate the need for clearer, tailored messaging to avoid reinforcing homophobic stereotypes; many high-risk individuals relied on social media for information, rather than government sources [17]. Research also shows that while nearly half of U.S. MSM adopted prevention strategies, there remains a gap in understanding the motivations behind these behavioral changes, including vaccination rates, and the most effective pathways for achieving positive outcomes [18]. Additional research into these areas, alongside the physical and psychosocial burdens of disease, is essential for informing future public health strategies and continued responses to mpox, ultimately fostering community trust and improving health outcomes.

In addition to addressing psychosocial health and evaluating public health responses, there has been minimal research focusing on the lived experiences of the MSM community during the mpox outbreak. Published research has predominantly focused on quantitative approaches, and has prioritized clinical and epidemiological endpoints. However, in order to enhance our understanding of the outbreak and inform future response, it’s necessary to capture the experiences of the MSM community, including their reception of public health messaging, experience accessing prevention and care, and psychosocial impacts of the mpox outbreak.

## Methods

### Participant Population

The study team recruited sexually active MSM aged 18 and older through two primary methods. Potential participants were contacted via text, call, or email from an existing database maintained by the UCLA Center for LGBTQ+ Advocacy, Research & Health, consisting of individuals who had previously agreed to be contacted for future research opportunities. Flyers were distributed at locations frequented by MSM in Los Angeles County, including bars, clubs, and HIV/STI clinics. The sampling strategy was convenient and purposive, prioritizing diversity in HIV status, race, ethnicity, and mpox vaccination history. While the goal was to enroll 30 participants, data collection stopped after 21 interviews when thematic saturation was reached.

### Data Collection

Between November 2023 and March 2024, enrolled participants (N = 21) completed a one-hour, semi-structured qualitative interview conducted in English via zoom. To maintain privacy, both interviewers and participants joined from secure locations with sufficient access to wireless signal. The interviews explored several key topics, including mpox-related knowledge, mpox experiences, mpox vaccination experiences, perceptions of mpox-related public health messaging, risk compensation, and psychological impacts of the mpox epidemic. Interviews were audio recorded. Prior to completion of the qualitative interview, enrolled participants completed a brief quantitative survey that assessed sociodemographic characteristics, mpox-related knowledge, perceived risk and concern related to mpox, and other health-related factors.

### Data Analysis

The zoom transcriptions from semi-structured interviews were validated against the original audio file by two authors (EAY, KA) to ensure accuracy, correct any discrepancies, and redact any personally identifiable information. Transcripts were analyzed in Dedoose (Version 9.0.17) by research staff (CGW, LR, KA) and the principal investigator using content analysis [19, 20]. Initially, an inductive coding approach was employed to analyze the data, with codes corresponding to the aforementioned interview guide domains. Following preliminary examination of the data, the research team adopted an emergent approach, allowing for identification of thematic categories distinct from our pre-specified domains. Coders iteratively discussed transcripts and emerging themes, resolved coder disagreements, and revised the codebook. We aimed to incorporate reflexivity and address potential researcher bias by dually prioritizing a high level of inter-coder reliability (Cohen’s Kappa > 80%) and respecting reasonable disagreement based on the diverse lived experiences of the research team [21]. The coders also employed eight qualitative research process-based measures of inter-coder reliability that have been previously proposed [22].

### Ethics and Informed Consent

Certified exempt IRB approval for the study was obtained at both UCLA and Brown University. The research team shared a detailed Study Information Sheet for participants to review and ask any questions before enrollment in the study. After completion of quantitative survey and semi-structured interview, participants were compensated $60.

## Results

Most participants self-identified as gay (90%), followed by bisexual (5%), and pansexual (5%). One-third (33%) were living with HIV. The mean age of participants was 40 years (SD = 15), and approximately half identified as Black/African American or mixed-race (48%), followed by White (38%), “Other” (10%), and Asian (5%). Approximately half of participants (48%) identified as Hispanic/Latinx. Over half (57%) reported receiving at least one dose of the mpox vaccine, while 38% reported receiving the two recommended doses for maximum protection. One participant indicated a previous diagnosis of mpox. See Table 1 for additional sociodemographic characteristics and other mpox- and health-related variables.

**Table 1:** Self-reported sociodemographic characteristics, mpox vaccination status, and other health related factors among gay, bisexual and other men who have sex with men in Los Angeles (N = 21)

	Total (N = 21)
	**Mean (SD)**
*Age*	39.90 (15.22)
	**N (%)**
*Current sexual identity*	
Gay	19 (90.48%)
Bi/Pansexual	2 (9.52%)
*HIV status*	
Negative	14 (66.67%)
Positive	7 (33.33%)
*Hispanic/Latinx ethnicity*	
Yes	10 (47.62%)
*Race*	
Black or African American	9 (42.86%)
White or Caucasian	8 (38.10%)
Asian	1 (4.76%)
Mixed Race/Other	3 (14.29%)
*Educational attainment*	
Some high school or less	2 (9.52%)
High school graduate or GED	3 (14.29%)
Completed some college	3 (14.29%)
Associate degree	5 (23.81%)
Bachelor's degree	5 (23.81%)
Master's degree or higher	3 (14.29%)
*Employment status*	
Yes	15 (71.43%)
No	6 (28.57%)
*Household annual income before taxes*	
Less than $25,000	6 (28.57%)
$25,000 to $34,999	5 (23.81%)
$35,000 to $49,999	5 (23.81%)
$50,000 to $74,999	1 (4.76%)
$75,000 to $99,999	2 (9.52%)
$100,000 or more	2 (9.52%)
*Prior mpox vaccination*	
Not vaccinated	8 (38.1%)
One dose	4 (19.05%)
Two doses	8 (38.10%)
“Don’t know”	1 (4.76%)
*Prior mpox diagnosis*	
Yes	1 (4.76%)
*Self-reported eligibility for mpox vaccination*	
Yes	14 (66.67%)
No	1 (4.76%)
Don’t know	5 (23.81%)
Decline to answer	1 (4.76%)
*Prior COVID-19 vaccination*	
Not vaccinated	3 (14.29%)
One dose	3 (14.29%)
Two doses	5 (23.81%)
Two doses and one or more booster shots	10 (47.62%)
*Insurance type*	
Private insurance	5 (23.81%)
Medicaid/medi-cal	11 (52.38%)
Medicare	3 (14.29%)
None	2 (9.52%)
*Seen a healthcare provider (past 12 months)*	
Yes	17 (80.95%)
No	3 (14.29%)
Decline to answer	1 (4.76%)
*Bacterial STI diagnosis (past 12 months)*	
Yes	9 (42.86%)
No	12 (57.14%)

Below, we present the qualitative themes identified through analysis of 21 transcripts, supported by corresponding quantitative results from the brief surveys. Selected illustrative quotes are provided in Table 2.

**Table 2:** Illustrative Participant Quotes

Section	Domain	Selected quotes (participant characteristics)
1	Mpox-related knowledge	*Symptoms:* “Obviously, there's like body aches, and, like, you can get really, like, physically ill. I know some people have, like, complained about like vomiting, and like not being able to like digest food and stuff. But also, you get like, you know, like chicken pox? But monkeypox? Which is like little tiny nodules that sound and look really painful, um, and you can get them all over your body and get them inside your body, too.” (Black/African American, Hispanic/Latino, 31-year-old, HIV seronegative) “Well, from what I remember I think like rashes. And I think like fevers or something like that, if I'm not mistaken.” (Hispanic/Latino, 34-year-old, HIV seronegative) *Knowledge source:* “I got most of my information from off the apps and not really from like the news and stuff like that…social media and dating apps.” (Black/African American, 32-year-old, HIV seronegative)
2	Psychological impact	“It was a difficult thing just trying to heal, and it wasn't visible. But you still very much were dealing with it” (Black/African American, 35-year-old, HIV seronegative) “They said they felt terrible. They had a lot of self-image issues going on at the time.” (White, 33-year-old, HIV seropositive) “For me it was heavy emotionally at the time. I think it was around the summer, so everyone was trying to just have fun go out travel, and then we got hit by with this little epidemic of mpox, so it was hard for me to see friends in my community go through this.” (White, 33-year-old, HIV seropositive) “I know it did cause a lot of concern, or maybe a lot of panic in the community.” (Black/African American, 32-year-old, HIV seronegative)
3	Comparisons to the HIV pandemic	“Cause when it [mpox] came out, like a lot of like LGBT men were like very like frightened about it. You know they were talking about the days like, you know the AIDS, you know. You know, that day. They were comparing it to that time.” (Black/African American, 34-year-old, HIV seronegative) “Like HIV. They didn’t take it seriously back in the beginning, because they said, ‘Oh, it's just for gay people.’ Okay, well, if they said anybody could be affected by it, I think we would have had a cure by now. Cause it took them years to even think about researching the AIDS, HIV virus. Because it didn’t- it didn’t concern them. It didn’t affect them at the time, so yeah…They should’ve had a blanket statement saying, ‘Okay, this is for- this is for anybody, it affects anybody.’” (Black/African American, 66-year-old, HIV seropositive) “Well, you know, I think it was kind of discrimination with the gay people cause a lot of people they were saying like, ‘Oh, no, that one is only for gay people, you know, this is people who's getting the HIV, who's getting all those monkey pox. This is only because a lot of people they have, you know, sex men with men or women with women stuff like that.’ (White, 40-year-old, HIV seropositive) “Like I remember when the HIV pandemic in the 80s, the late 80s. It was all about the gay community. ‘HIV is only for gay people.’ Uh, no. It's not. Everybody can get it, and let's be real, you know. And the same thing with the monkeypox.” (White, 55-year-old, HIV seropositive)
4	Mpox vaccination experiences	*Facilitators* “So at first, I was not going to get the vaccination at all. But then I went to go see my primary care physician and my primary care doctor, uh, because I'm HIV positive, that he thought it might be a good idea for me to get the vaccination.” (Black/African American, Hispanic/Latino, 30-year-old, HIV seropositive) “Participant: Yeah, most especially the family, actually. They were kind of encouraging me to at least take a dose. I decided to give it a try.” (Black/African American, Hispanic/Latino, 29-year-old, HIV seronegative) “Well, I was like dating more at the time, and I knew it was like common among gay guys. So I was like, ‘All right, like I should probably take it the vaccine sooner than later,’ you know, cause I don't want to get it [mpox].” (Hispanic/Latino, 20-year-old, HIV seronegative) “Coming out of this like COVID situation, where, like everybody, was like desperate for some sort of defense against it. And then here's this other thing, that not only is it kind of new to our population, but there is a vaccine that, like had already existed, for, like at least like a decade. I started seeing, like, these different flyers right on Instagram and then on Twitter. And I was like, ‘I need to just go to one and get in line and hope for the best.’” (Hispanic/Latino, 20-year-old, HIV seronegative) “I just wanted to be safe. Cause I was seeing the pictures of people with mpox. Oh, my God, that was horrible! So, if it’s a vaccine, and I like to think I am open to vaccines that have been tested so, so that was the best way to get my immune system against it.” (Black/African American, 28-year-old, HIV seronegative) *Barriers* “I gave the Department of Public Health my information, and they contacted me eventually. But because they kind of lagged, as state services often do, I decided to just go on my own and see if I could get it. And I did. I think a lot of people had that same story.” (Mixed-Race, Hispanic/Latino, 31-year-old, HIV seronegative) “I don't like shots. If I don't have to get one, I’ll do whatever it takes, but I- I don't want a shot. I can’t even handle a blood test.” (Hispanic/Latino, 63-year-old, HIV seropositive) “You know, I'm lucky I have a car. So even though the that place was far, I have a car, so drove down on a Sunday morning….For people who don't have cars, it might have been a lot more difficult to get a bus or uber or something.” (White, 65-year-old, HIV seropositive) “The spots were filling up so quick. So even if we tried, it was just like, ‘Oh, sorry, there are no spots available’ or something. And then time passed, and we just haven't gotten the vaccine.” (Asian, 26-year-old, HIV seronegative)
5	Community mobilization	“I had heard about it when everyone was talking about it. I'm on Facebook and Instagram, so you know, I have a lot of gay friends, and that's what people are talking about and posting warnings. And you know people doing their own public service. ‘Hey, this is going on’” (White, 65-year-old, HIV seropositive) “It gave me joy when I was able to work with my community and the rollout of that vaccine. I was working at a clinic at the time, and we were able to get a resource for the vaccinations, and I was able to get a lot of the word out to many of my friends and family about ‘Hey You know, there's this new resource. If you're interested, let me know like I can hook you up’” (White, 33-year-old, HIV seropositive) “I remember seeing little pop-ups of vaccinations at the bar, you know at the gay bar, at the club, outside of small businesses who were nice enough to, you know, to let us table outside of their businesses. The mobilization aspect of it felt really good as a community coming together to try to help vaccinate the community.” (White, 33-year-old, HIV seropositive)
6	Reactions to mpox health messaging	“I felt it was important and necessary [for messaging to focus on GBMSM], and I'm actually kind of glad that the public messaging about it was very direct.” (Mixed-Race, Hispanic/Latino, 31-year-old, HIV seronegative) “I was happy that like they were like warning gay guys. But I also felt like, if you say it's only spreading or it's spreading majority among gay guys, like some people would start to blame like gay guys for spreading it. But overall, I think, like the good outweighs the bad. And I was happy that like, yeah, there was like public health awareness around it.” (Hispanic/Latino, 20-year-old, HIV seronegative) “Well, I would have preferred it with this if they would have said it affects anybody. Instead of singling out gay people or Black people, whatever. It affects anybody. Then, maybe I think people would take more seriously.” (Black/African American, 66-year-old, HIV seropositive) “It’s one of them things that people think they only got, only get it if they if you're gay. It was like, people thought it didn’t- ‘Oh, it doesn’t apply to me, I mean, it's just a gay person’s disease.’” (Black/African American, 35-year-old, HIV seronegative)
7	COVID-19 comparisons	“I was actually quite impressed with how well information was getting out there. Especially considering how you know, during the Trump years, that during the pandemic information wasn't getting out, and he made nothing of it. Said ‘Oh it's just the flu, and it'll be gone’ and then it turned into a disaster. But mpox seemed like as soon as it got out there, information was flying left and right. And then, just a short time later, vaccines became available. I was impressed by all that.” (White, 65-year-old, HIV seropositive) “I told my parents I was gonna get the vaccine cause at the time we were all like ‘Wait’ cause we were coming out of the direct post pandemic COVID era, and a lot of people were very confused about viruses they've never heard before, and my mom was very confused. I just remember it being really scary, and I remember talking to my family about it and reading about it, doing my own research.” (Mixed-Race, Hispanic/Latino, 31-year-old, HIV seronegative) “The second dose itched more than the first dose, but I didn't have any type of flu-like symptoms like you get after, say, a COVID or a flu shot.” (Hispanic/Latino, 29-year-old, HIV seronegative) “Coming out of this like COVID situation, where everybody was desperate for some sort of defense against it. And then here's this other thing [mpox], that not only is it kind of new to our population, but there is a vaccine that, like had already existed, for, like at least like a decade. I was like, ‘Yeah, no, let’s just-’ I don't wanna go through like horrific pain at all. If I can avoid it, I will.” (Mixed-Race, Hispanic/Latino, 31-year-old, HIV seronegative) “It was the same way with COVID, you know. When COVID first came around, people were getting sick. Vaccines were barely starting to roll out. People had to wait. So it was kind of that all over again in some way.” (White, 33-year-old, HIV seropositive)
8	Behavior changes	“Everyone was just on such high alert, and it was the summertime. People weren't going out as much. People were afraid to – like the hookup culture was afraid to be doing their thing. It was different and weird.” (Black/African American, 34-year-old, HIV seronegative) “I stopped going out completely.” (White, 55-year-old, HIV seropositive) “At the time, I didn't really wanna like fuck with people, cause I was afraid. After I got my vaccine dose – I got both doses – I was like, ‘Okay, I feel a lot better.” (Mixed-Race, Hispanic/Latino, 31-year-old, HIV seronegative) “Yeah, I feel like people were still on the lookout, you know. I remember friends screening their sexual partners. You know, both like, just casually and physically. I think at the time I still knew people who had still hadn't gotten their second [dose]. Yeah, they were still a little bit more concerned, even just last summer.” (White, 33-year-old, HIV seropositive) “I go to pride every year. And so I skipped pride last year because of that [mpox].” (Black/African American, 34-year-old, HIV seronegative)
9	“Return to normal”	“The hype has kind of gone down. I feel like a lot of people by this point have either forgotten about it or have taken steps towards protecting themselves.” (White, 33-year-old, HIV seropositive) “It’s basically nonexistent. It doesn't even come up in conversation anymore.” (Black/African American, 32-year-old, HIV seronegative) “I don't think about it [mpox] at all. I mean, if you know, there's some sort of resurgence in there like COVID changes, maybe mpox was to change in a new outbreak. But I don't stay at nights worrying about that.” (White, 65-year-old, HIV seropositive)
10	Current concerns about mpox	“No, I'm still concerned. When a pandemic is at its peak, yes, everybody's gonna freak out. But when the numbers go down people start doing risky things again… we're not at the peak no more, but I still think it could possibly– I still look it up. Monkeypox, it’s still out there. So for me, I'm still gonna stay safe. Somebody told me, ‘I'm not really worried about it’, I was like, ‘Well, you should,’ because, you know, this is something serious. People have been diagnosed, and I heard it's very worse for people that have HIV.” (Black/African American, 34-year-old, HIV seronegative) “Well, you know, honestly, I'm still kind of scared in a little bit of a way, because I kinda feel like that just because you have the vaccination that probably don't mean nothing.” (Black/African American, Hispanic/Latino, 30-year-old, HIV seropositive)

### Mpox-Related Knowledge

**Fig. 1:**
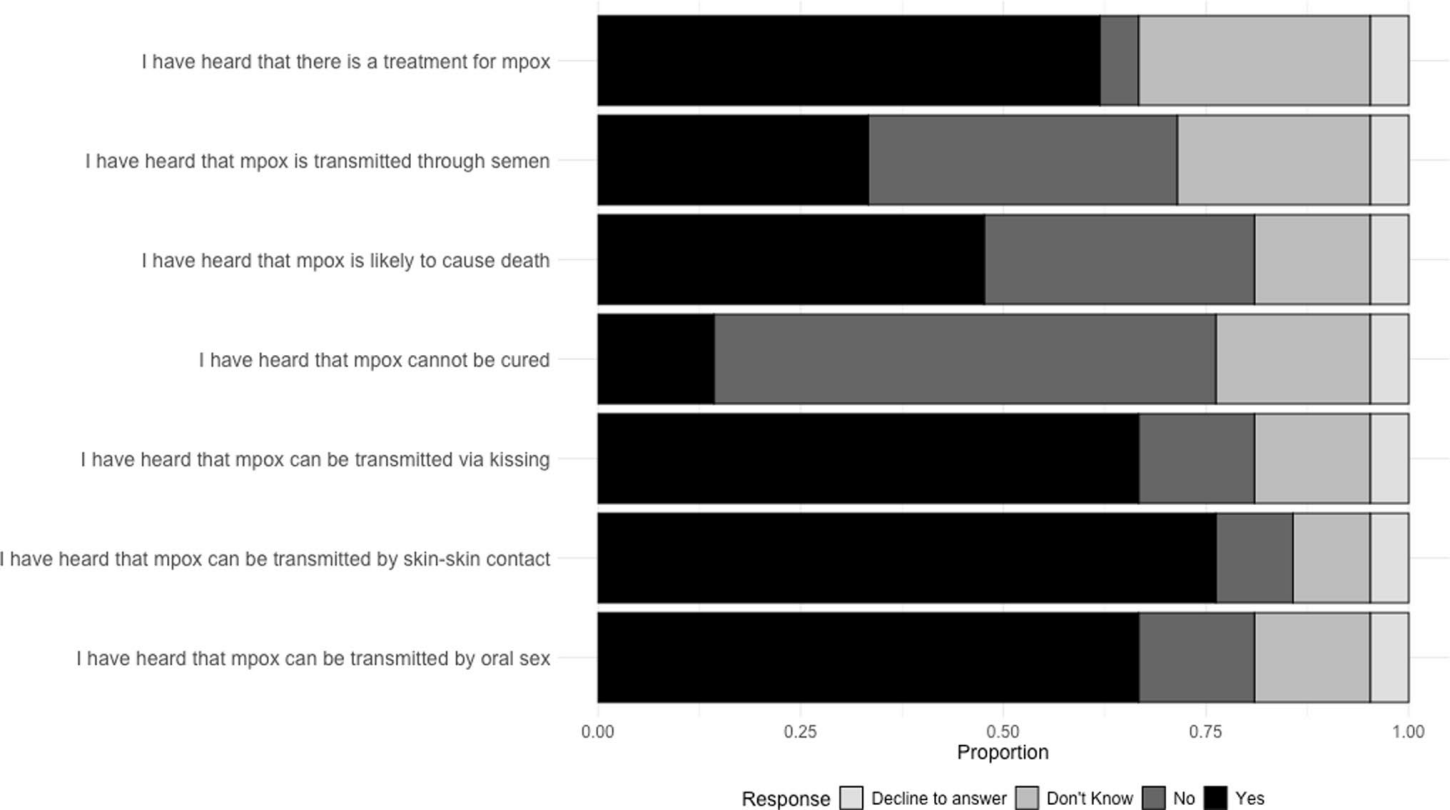
Participant knowledge related to mpox transmission routes, treatment, and outcomes

Most participants were familiar with symptoms associated with mpox infection, particularly the presence of pox-like lesions and flu-like symptoms (See Table 2, Sect. 1). Participants reported hearing that mpox could be transmitted by skin-to-skin contact (76%), kissing (67%), oral sex (67%), and semen (33%) **(**Fig. 1). Regarding mpox treatment and outcomes, 62% were aware that there was a treatment for mpox, 14% had heard that mpox cannot be cured, and nearly half of the participants (48%) believed that mpox was likely to cause death (Fig. 1).

### Mpox Experience

One participant described his firsthand experience with mpox, including being misdiagnosed by a healthcare provider before receiving an accurate diagnosis through participation in an HIV pre-exposure prophylaxis (PrEP)-related research study. Another participant reported knowing someone who died from mpox, while several others shared secondhand experiences of mpox as conveyed by their social connections and sexual partners.

### Psychological Impact

Feelings of isolation and embarrassment related to the process of mpox diagnosis and treatment were mentioned in both first- and secondhand accounts (Table 2, Sect. 2). Additionally, poor self-image resulting from skin lesions and scarring was reported, with one secondhand account detailing a man with mpox who stayed home from work for an extended period beyond the isolation requirements due to his concerns about his physical appearance. Beyond the individual-level psychological impacts of the Los Angeles mpox epidemic, many participants described the community-level distress, fear, and sense of responsibility to keep their community informed and protected (Table 2, Sect. 2). Other participants compared their emotional response with how they felt during the onset of the HIV/AIDS epidemic (Table 2, Sect. 3).

### Mpox Vaccination

During qualitative interviews, participants shared their experiences with mpox vaccination. The most commonly reported reason for seeking mpox vaccination was fear of infection during the initial outbreak, followed by a desire for personal and community protection. Other facilitators of vaccination included getting vaccinated with friends, the accessibility of vaccination at venues frequented by MSM (e.g., outside of gay clubs and bathhouses), a personal belief in the efficacy of vaccines, and the availability of a pre-existing vaccine. Vaccinated participants reported that pain, itchiness, and redness following vaccination were “frustrating” to deal with, but they felt “it was certainly worth not getting mpox.” Noted barriers to mpox vaccination included long wait times (> 2 h), the need to take off work, lack of transportation to vaccination sites, mistrust of vaccines, and “a confusing vaccine rollout.” One participant was unaware that a vaccine for mpox was available. Multiple participants reported dissatisfaction with the pace of vaccine rollout and finding information more quickly about where to get vaccinated by word of mouth, social media, and dating apps (Table 2, Sect. 4).

### Community Mobilization

Several participants attributed their knowledge/awareness about mpox and mpox vaccination to efforts of MSM community organizers tabling outside of venues and being vocal on social media and dating apps (Table 2, Sect. 5). Some participants reported directly being involved with dissemination of mpox and vaccination knowledge to their community.

### Reactions to Mpox Health Messaging

Participants generally thought messaging around mpox and mpox vaccination was effective (Table 2, Sect. 6). Others made comparisons to HIV messaging, saying it caused “concern” and “panic” in the community, contributing to the stigmatization of MSM populations (Table 2, Sect. 3).

### Comparisons to COVID-19

Several participants referenced the COVID-19 epidemic when discussing their experiences with mpox and mpox vaccination. Participants generally perceived mpox messaging and timing of vaccine rollout to be more effective and efficient than the COVID-19 response, which participants thought had a lot of misinformation. Restricted initial vaccine eligibility for mpox vaccination was compared to COVID-19 vaccination eligibility. Participants understood the goal was to vaccinate those with the highest mpox risk, as with COVID-19 (Table 2, Sect. 7). Participants thought mpox vaccine side effects were “not as bad” as those from COVID-19 vaccination.

### Behavior Changes

During the initial mpox outbreak in Los Angeles, some participants reported minimizing going out and sexual meetups from dating apps, initiating conversations about mpox, STIs, and HIV with new sexual partners (“Are you clean?”), visual checks for rashes with potential partners, and wearing long sleeves at the gym to reduce risk of mpox during the initial outbreak in 2022 (Table 2, Sect. 8).

### Current Mpox Concern

**Fig. 2:**
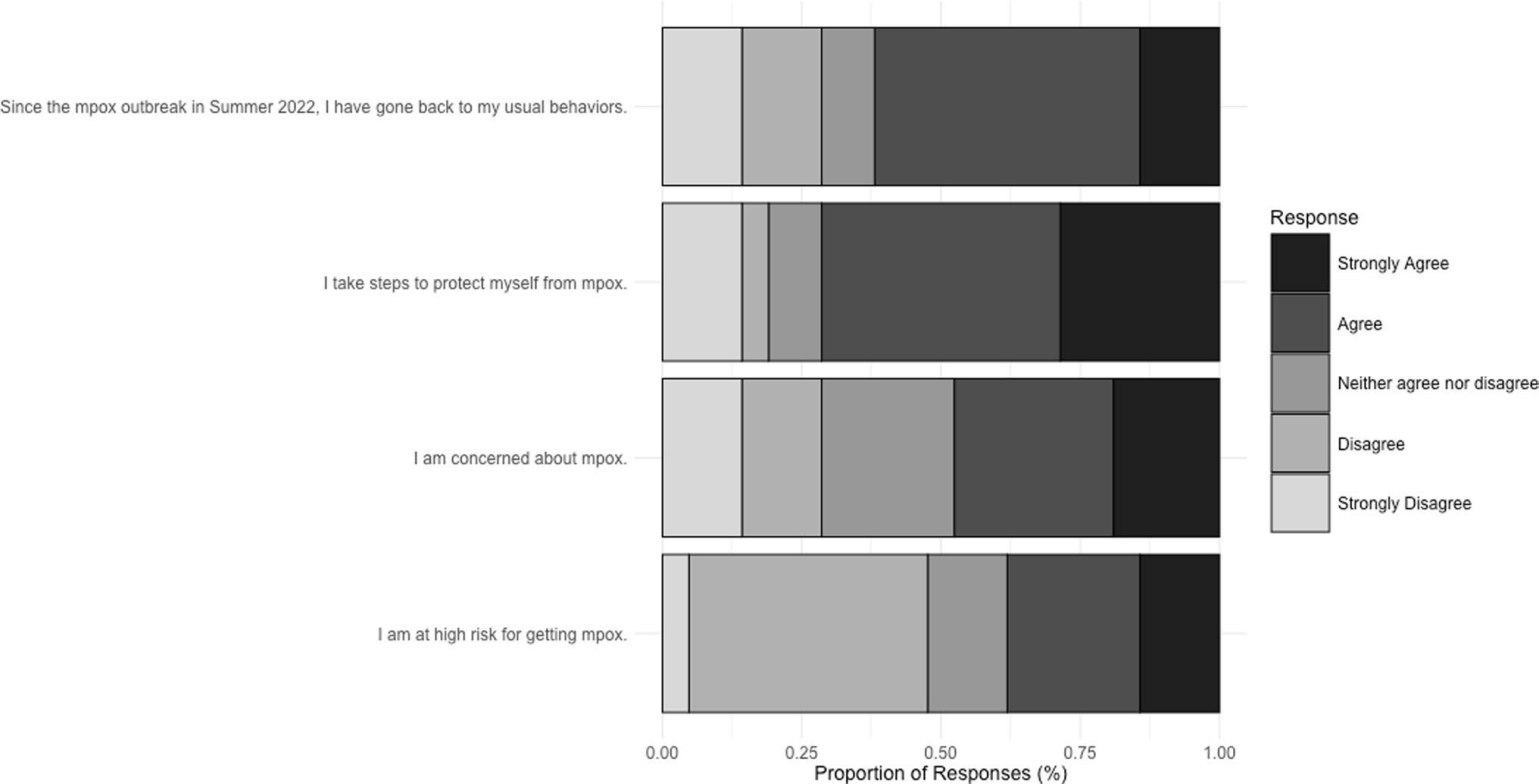
Perceived level of risk and concern for mpox and engagement in protective behaviors

Participant responses to questions related to mpox current level of concern, perceived level of risk, and engagement in protective behavior are summarized in Fig. 2. Those who were vaccinated generally felt safer and more prepared for a future mpox outbreak after receiving both doses. Moreover, since the initial outbreak, most participants report returning to their pre-outbreak activities (Fig. 2; Table 2, Sect. 9). Even after vaccination, some participants remained concerned about mpox, particularly for people living with HIV (Table 2, Sect. 10).

## Discussion

In the current study, we used a mixed methods approach to learn more about the nuanced lived experiences of MSM during the 2022 mpox epidemic in Los Angeles and to better understand individual behaviors, perceptions of health messaging and programming, testing and treatment experiences, vaccination decisions, and the psychosocial impact that mpox had on them. In doing so, we aimed to provide actionable insights to enhance public health strategies and responses to future mpox outbreaks that leverage community resilience and support systems to achieve favorable health outcomes for all.

The study demonstrates that knowledge levels about the mpox epidemic varied. While most participants understood that mpox can spread through close or intimate contact, consistent with existing research [23–25], many participants also incorrectly believed that mpox is likely fatal and cannot be cured. The belief that mpox “cannot be cured” may reflect misunderstandings about treatment efficacy, particularly given the limited clinical evidence supporting the effectiveness of TPOXX (tecovirimat) [26]. This perception, while not entirely unfounded, may have contributed to heightened epidemic-related fear and mixed messaging around disease severity and available treatments at the time of survey. These mpox-related knowledge gaps may reflect a lack of clarity in mpox messaging, and mirror knowledge challenges observed in other public health crises, including HIV and COVID-19 [26–29]. Findings highlight that participants heavily relied on LGBTQ + community and peer networks as sources of information, which could be leveraged for future interventions, but ensuring that accurate information is provided is important.

In our study, psychological impacts emerged as the most common theme in participants’ descriptions of their mpox-related experiences. Participants reported substantial emotional distress, including fear and panic surrounding mpox acquisition, and self-image issues associated with symptoms. Participants reported withdrawal from social life and community-level distress, partly due to the felt responsibility of educating and keeping their community safe. This further supports growing research around the psychological impact of the mpox epidemic on both an individual and community level, inclusive of those directly impacted by mpox and those at higher risk of acquisition [30, 31]. The mpox epidemic directly heightened experiences of depression, anxiety, and stress among a marginalized group that already experiences a disproportionate mental health burden [31]. Some participants compared the mpox epidemic to their experiences with the early days of the HIV/AIDS epidemic, when MSM experienced significant fear and stigma, and when access to dedicated HIV prevention programs and resources were limited. Programmatic response to mpox should integrate support for psychological health not only for those directly experiencing symptoms, but also at a community level for collective impact [31–33].

Participants predominantly reported protective behavioral changes during the mpox epidemic. Existing literature demonstrates that MSM behavior change during the mpox outbreak varied. Results from the annual American Men’s Internet Survey (AMIS) found that about half of all respondents reported adopting prevention strategies [18]. However, the reasons behind these changes were not explored, providing an opportunity for research in this area. In the current study, intense fear of mpox acquisition among MSM was the primary motivator for behavior modification. Participants described their mpox-related behavioral changes primarily in sexual or “hook-up” contexts, including decreases in the frequency of sexual encounters, increased conversations about STI and mpox risk, and engaging in visual checks for lesions. Outside of these contexts, participants reported modifying their appearance (e.g., wearing long sleeves at the gym) and limiting or completely stopping attendance of social events, consistent with other literature [18]. A subset of individuals described lower perceived concern and risk of mpox despite engaging in higher-risk behaviors (sexual activity, attending crowded social events, etc.). This discordance could be attributed to mpox misinformation or “epidemic fatigue” because of constant navigation of COVID-19 and in some cases, HIV prevention, among MSM, or alternatively due to perceived protection after mpox vaccination. Additionally, lower perceived risk may be in part facilitated by sexual partner hookup apps, like Grindr, which allowed for users to add their mpox vaccination status, along with COVID-19, on their profiles. Over time, most participants resumed pre-outbreak activities, reflecting a “return to normal” concurrent with a decrease in mpox cases and increase in vaccination uptake. At the time of data collection, well after the initial outbreak in 2022, participants reported a decrease in mpox concern and self-perceived risk of mpox. However, it was notable that participants reported they continued to take steps to protect themselves from mpox, suggesting a long-term adoption of meaningful prevention strategies.

Overall mpox vaccination uptake in U.S. metropolitan areas was low [34]. Vaccination uptake is pivotal in controlling an mpox outbreak, however data from the U.S. CDC indicates 1- and 2-dose vaccination coverage only reached an estimated 37% and 23%, respectively, among persons at risk [34]. Furthermore, 2022 mpox vaccine administration data in the U.S. highlighted important health care disparities, with the majority of mpox cases occurring among Black and Hispanic/Latinx individuals, but these groups constituting a minority of those accessing a vaccine [35, 36]. The results of this study can help contextualize both facilitators and barriers to mpox vaccination among diverse, metropolitan, MSM. In the interviews, participants cited health care recommendation, vaccine access and integration into community spaces, and the availability of a pre-established vaccine as motivators for vaccination. Conversely, the barriers inhibiting vaccine uptake beyond the perceived slow rollout, included access issues (long lines, availability during working hours, distance to vaccine providers) and difficulty accessing vaccine information, eventually found through social media, word of mouth, and dating apps. Trusted sources of information have historically influenced vaccine motivations, and this continued for mpox [23, 29]. Trusted health care personnel, especially for MSM who may already be engaged in care for HIV treatment or prevention, serve as a primary facilitator not only for vaccine uptake, but also for vaccine trust, which is particularly important with growing vaccine hesitancy in the U.S. Outside of health care settings, participants perceived the community mobilization efforts to establish vaccine “pop-ups” in trusted spaces to have been effective, which has previously been established [37], addressing interconnected facilitators of access, trust, and comfortability vital for health emergencies impacting historically marginalized groups.

Public health communications and community engagement were a key component of the U.S. mpox response. The U.S. CDC tested messages and participated in community engagement and listening sessions to develop mpox-related communications and disseminate them via dating apps and other MSM media [2]. However, to the best of our understanding, there are currently no peer-reviewed papers evaluating the reception, acceptability, or optimizations of these materials and approach. Our findings and others highlight that most participants received mpox-related information from (specifically Tik Tok, Twitter, Facebook, and Instagram), dating apps, and word of mouth, rather than government sources [17, 23, 31]. This also aligned with previous research from the COVID-19 epidemic [29], suggesting these community-based information pathways are trusted and can be leveraged in future outbreaks to quickly and effectively disseminate guidance around disease prevention, access to resources, and knowledge around treatment and vaccine updates. While messaging around mpox vaccine access was generally well received in comparison to COVID-19, participants raised concerns about stigma and panic, drawing parallels to early HIV/AIDS communication. They emphasized the need for clear, non-stigmatizing messaging that avoids homophobic stereotypes. Community-led dissemination of trusted, affirming information remains important in the context of the current administration, where community trust, support, and resilience are vital when LGBTQ+ health and human rights protections are under attack.

Our study had several limitations. The study data were collected after the initial mpox outbreak in Summer 2022, potentially impacting participants’ ability to accurately recall their experiences during this time. Some participants in the study described being primarily in monogamous relationships during the initial mpox outbreak, which could have reduced their level of risk for mpox infection. However, studies show that sexually transmitted infection risk, which is also related to risk for mpox, is non-negligible among persons self-reporting monogamous relationships [38, 39]. The sample is non-random and was predominately recruited from a longitudinal research study focused on HIV/AIDS, which may limit generalizability of health-related behavior and opinions. Although substantial for qualitative data, the smaller sample size limits the ability for powered quantitative comparisons.

The findings from our study are particularly relevant given the current political climate, where cuts to federal public health funding in the U.S. under the Trump administration have stalled key infrastructure upgrades, including efforts to modernize outdated data systems that reportedly hindered responses to both COVID-19 and mpox outbreaks [40]. Additionally, the current administration has rolled back protections for LGBTQ+ individuals, further eroding trust in government-led health efforts among marginalized communities. Community-based, culturally responsive approaches to messaging and outbreak prevention are increasingly essential to ensure an effective response to future outbreaks, particularly when governmental support systems are strained or undermined.

## Conclusions

This study documents a group of gay, bisexual, and pansexual men’s experience with the mpox epidemic, experiences with mpox vaccination, and the impacts of the mpox outbreak and public health response on HIV mixed-serostatus MSM in Los Angeles County. The individual and community-level psychological impacts of mpox are concerning; however, our findings highlight the resilience and ability of the MSM community in LA to mobilize to increase awareness about mpox and promote vaccination uptake, resulting in feelings of preparedness for future outbreaks. This study highlights an opportunity for behavioral intervention in future outbreaks to mitigate distress and anxiety, especially for communities at higher risk of severe illness and death, including people living with HIV who are immunocompromised. The results prompt a need to better understand vaccination choices among the MSM community, including perceived risk, formal vaccine rollout, vaccine accessibility, and medical trust. Through understanding the decision-making process, as well as how information pathways (social media, word of mouth, dating apps) promote or inhibit this process, prevention efforts can address barriers and improve facilitators to vaccination uptake, enhancing future campaigns and public health policies.

## Data Availability

Due to the sensitive nature of topics covered and risk of potential identifiability of participants despite anonymization, the data presented in this paper are not available for sharing.

## Abbreviations

WHO: World Health Organization
MSM: Gay, bisexual, and other men who have sex with men
U.S.: United States
CDC: Centers for disease control and prevention
PLWH: People living with HIV
